# Global Loss of Core 1-Derived *O*-Glycans in Mice Leads to High Mortality Due to Acute Kidney Failure and Gastric Ulcers

**DOI:** 10.3390/ijms23031273

**Published:** 2022-01-24

**Authors:** Riku Suzuki, Yuki Nakamura, Rikako Koiwai, Sayaka Fuseya, Yuka Murakami, Kozue Hagiwara, Takashi Sato, Satoru Takahashi, Takashi Kudo

**Affiliations:** 1 Department of Anatomy and Embryology, Faculty of Medicine, University of Tsukuba, Tsukuba 305-8575, Japan; iapvb.202@icloud.com (R.S.); ynakamura593@gmail.com (Y.N.); hirametama37@gmail.com (R.K.); xxxisep3@gmail.com (S.F.); s1811791@s.tsukuba.ac.jp (Y.M.); 2 Ph.D. Program in Human Biology, School of Integrative and Global Majors, University of Tsukuba, Tsukuba 305-8575, Japan; 3 Doctoral Program in Biomedical Sciences, Graduate School of Comprehensive Human Sciences, University of Tsukuba, Tsukuba 305-8575, Japan; 4 College of Medical Sciences, School of Medicine and Health Sciences, University of Tsukuba, Tsukuba 305-8575, Japan; 5 Department of Life Science and Biotechnology, Cellular and Molecular Biotechnology Research Institute, National Institute of Advanced Industrial Science and Technology, Tsukuba 305-8565, Japan; k.hagiwara@aist.go.jp (K.H.); takashi-sato@aist.go.jp (T.S.); 6 Laboratory Animal Resource Center in Transborder Medical Research Center, Faculty of Medicine, University of Tsukuba, Tsukuba 305-8575, Japan

**Keywords:** *O*-glycosylation, acute pancreatitis, renal failure, gastric ulcer

## Abstract

The core 1 structure is the major constituent of mucin-type *O*-glycans, which are added via glycosylation—a posttranslational modification present on membrane-bound and secretory proteins. Core 1 β1,3-galactosyltransferase (C1galt1), an enzyme that synthesizes the core 1 structure, requires Cosmc, a C1galt1-specific molecular chaperone, for its enzymatic activity. Since *Cosmc*-knockout mice exhibit embryonic lethality, the biological role of core 1-derived *O*-glycans in the adult stage is not fully understood. We generated ubiquitous and inducible CAGCre-ER^TM^/*Cosmc*-knockout (iCAG-Cos) mice to investigate the physiological function of core 1-derived *O*-glycans. The iCAG-Cos mice exhibited a global loss of core 1-derived *O*-glycans, high mortality, and showed a drastic reduction in weights of the thymus, adipose tissue, and pancreas 10 days after *Cosmc* deletion. They also exhibited leukocytopenia, thrombocytopenia, severe acute pancreatitis, and atrophy of white and brown adipose tissue, as well as spontaneous gastric ulcers and severe renal dysfunction, which were considered the causes underlying the high mortality of the iCAG-Cos mice. Serological analysis indicated the iCAG-Cos mice have lower blood glucose and total blood protein levels and higher triglyceride, high-density lipoprotein, and total cholesterol levels than the controls. These data demonstrate the importance of core 1-derived *O*-glycans for homeostatic maintenance in adult mice.

## 1. Introduction

**Figure 1:**
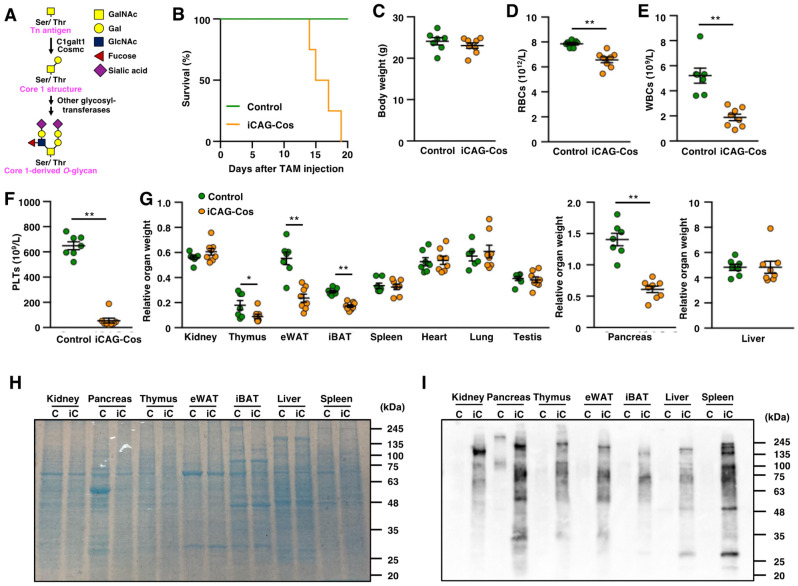
iCAG-Cos mice show high mortality and global loss of core 1-derived *O*-glycans. (**A**) Schematic showing biosynthetic pathway of core 1-derived *O*-glycans; (**B**) survival rate after tamoxifen (TAM) injection into control and iCAG-Cos mice; (**C**) body weights of control and iCAG-Cos mice on day 10; (**D**–**F**) number of red blood cells (RBCs), white blood cells (WBCs), and platelets (PLTs) on day 10 (control, *n* = 7; iCAG-Cos, *n* = 8); (**G**) relative organ weights of control and iCAG-Cos mice. iBAT, interscapular brown adipose tissue; eWAT, epididymis white adipose tissue. Tissues were collected on day 10, and organ weight was normalized by body weight. (**H**) The loading control of the *Helix pomatia* agglutinin (HPA) lectin blot shown with Coomassie brilliant blue (CBB) staining. (**I**) Expression of core 1-derived *O*-glycan in listed tissues using HRP-conjugated HPA lectin. C; control, iC; iCAG-Cos, * *p* < 0.05, ** *p* < 0.01.

Mucin-type *O*-glycosylation is posttranslational modification that occurs on membrane-bound and secretory proteins. The biosynthesis of mucin-type *O*-glycans starts with the initial addition of *N*-acetylgalactosamine (GalNAc) to the hydroxyl group of serine or threonine residues on proteins mediated by polypeptide *N*-acetylgalactosaminyltransferases to synthesize the Tn antigen (GalNAc-α-Ser/Thr structure; Figure 1A). Usually, galactose (Gal) is then transferred to GalNAc via core 1 β1,3-galactosyltransferase (C1galt1) [1]. This Gal-β1,3-GalNAc-α-Ser/Thr structure is called the core 1 structure, which is the major core structure of mucin-type *O*-glycans. Cosmc, the C1galt1-specific molecular chaperone, is essential for the activity of C1galt1 [2].

*C1galt1*- and *Cosmc*-knockout (KO) mice exhibit embryonic lethality with abnormal angiogenesis and hemorrhage in the brain and spinal cord, indicating that core 1-derived *O*-glycans are essential in the embryonic stage [3,4]. To investigate the physiological role of core 1-derived *O*-glycans in adults, we generated tissue-specific *C1galt1*- or *Cosmc*-KO mice. Previously, we reported that bone marrow cell-restricted *C1galt1*-KO mice exhibit severe thrombocytopenia due to abnormal terminal differentiation of megakaryocytes and proplatelet formation [5]. We also reported that core 1-derived *O*-glycans are necessary for the stable expression of T cell immunoglobulin and mucin domain-containing molecule 4 (Tim-4) glycoprotein in resident peritoneal macrophages and are important for the clearance of phagocytic debris [6]. Several studies have focused on the function of core 1-derived *O*-glycans in glomerular epithelial cells (podocytes) using podocyte-specific *C1galt1*- or *Cosmc*-KO mice [7,8]. The loss of core 1-derived *O*-glycans in these mice results in decreased stability of the glycoprotein podocalyxin, which leads to impaired glomerular function. Another study demonstrated that core 1-derived *O*-glycans are required for B cell development and regulate B cell homing [9]. These studies indicate the physiological importance of core 1-derived *O*-glycans for homeostasis in adult mice.

Unlike the *N*-glycosylation sites, the core 1-derived *O*-glycosylation sites do not have a consensus amino acid sequence, and both *C1galt1* and *Cosmc* are expressed in most tissues [10]. Therefore, it is expected that there are still numerous glycoproteins for which physiological functions are regulated by core-1 derived *O*-glycosylation. Although it has been demonstrated that glycoproteins with mucin domains, such as MUC and Tim family proteins, contain many serine or threonine residues it is not fully understood how the glycans on these proteins affect their functions [11,12]. Moreover, verification of a protein being modified by core-1 derived *O*-glycosylation in an individual is difficult and requires advanced equipment and expertise. Therefore, phenotypic analysis conducted using genetically modified mice is one of the most efficient ways to predict the glycosylated protein and its physiological functions.

In the present study, iCAG-Cos mice showed atrophy of adipocytes, acute pancreatitis, spontaneous gastric ulcers, and kidney impairment. Severe gastric ulcers and renal dysfunction were considered the causes of the high mortality observed in the iCAG-Cos mice. The long-term effect of core 1-derived *O*-glycan deficiency could not be analyzed due to the early death of iCAG-Cos mice. In addition, kidney dysfunction and pancreatitis posed difficulties in analyzing the physiological functions of other tissues. However, we suggest that the loss of core 1-derived *O*-glycans is a possible cause of acute pancreatitis and adipose tissue atrophy, although the detailed mechanism is still unknown. Moreover, we identified various cells that possess core 1-derived *O*-glycans in multiple organs by *Helix pomatia* agglutinin (HPA) staining. Further studies focusing on these phenotypes or organs are needed to deepen the understanding of the function of core 1 derived *O*-glycans in adults.

## 2. Results and Discussion

### 2.1. Ubiquitous Loss of Core 1-Derived O-Glycans Leads to High Mortality of Mice and Decrease in the Weight of Multiple Organs

To elucidate the function of core 1-derived *O*-glycans in adult mice, we generated conditional *Cosmc* KO (iCAG-Cos) mice using Cre driver mice that function with the tamoxifen (TAM)-inducible and widespread tissue-expressed CAG promoter. The day of the final TAM injection was defined as day 0. All mice died by day 19, whereas control mice showed no abnormalities due to TAM induction (Figure 1B). To avoid serious deterioration in the health of mice, we collected peripheral blood and organs on day 10. The iCAG-Cos mice had body weights similar to those of control mice (control, 24.10 ± 0.88 g; iCAG-Cos, 23.09 ± 0.57 g; Figure 1C). The number of red blood cells was slightly decreased in iCAG-Cos mice (Control, 7.86 ± 0.10 × 10^12^/L; iCAG-Cos, 6.57 ± 0.23 × 10^12^/L; Figure 1D). The white blood cell (WBC) counts were also decreased significantly (control, 5.21 ± 0.59 × 10^9^/L; iCAG-Cos, 1.89 ± 0.27 × 10^9^/L; Figure 1E). Previous reports have shown that T cell-specific *Cosmc*-KO mice and B cell-specific *Cosmc*-KO mice have fewer T cells and B cells in peripheral blood, respectively [9,13]. These studies imply that the reduced number of WBCs observed in iCAG-Cos mice is due to *Comsc* deletion in these cells. iCAG-Cos mice also showed a decreased number of platelets (PLTs; control, 649.92 ± 3.21 × 10^9^/L; iCAG-Cos, 5.44 ± 1.94 × 10^9^/L; Figure 1F). The decrease in platelet count was reported to be due to the defective terminal differentiation of megakaryocytes and increased clearance of PLTs by Kupffer cells in the liver [5,14].

By comparing organ weights normalized by body weight, the thymus, pancreas, interscapular brown adipose tissue (iBAT), and epididymal white adipose tissue (eWAT) weights were markedly reduced in iCAG-Cos mice, whereas other organ weights remained almost unchanged (Figure 1G). To confirm changes in the core 1-derived *O*-glycan structures of iCAG-Cos mice, we conducted HPA lectin blot analysis of tissue extracts from control and iCAG-Cos mouse organs. HPA lectin recognizes terminal α-GalNAc, the structure expected to be increased after the deletion of *Cosmc*. The pattern of protein bands obtained for the control and iCAG-Cos mice by Coomassie brilliant blue (CBB) staining was mostly similar for major organs, except for that of the pancreas (Figure 1H). Protein extracts from iCAG-Cos mice showed a higher HPA intensity for a variety of molecular weights, indicating that various proteins in these tissues are modified by core 1-derived *O*-glycans (Figure 1I).

### 2.2. iCAG-Cos Mice Exhibit Atrophy of the Thymus and Adipocytes and Acute Pancreatitis

**Figure 2:**
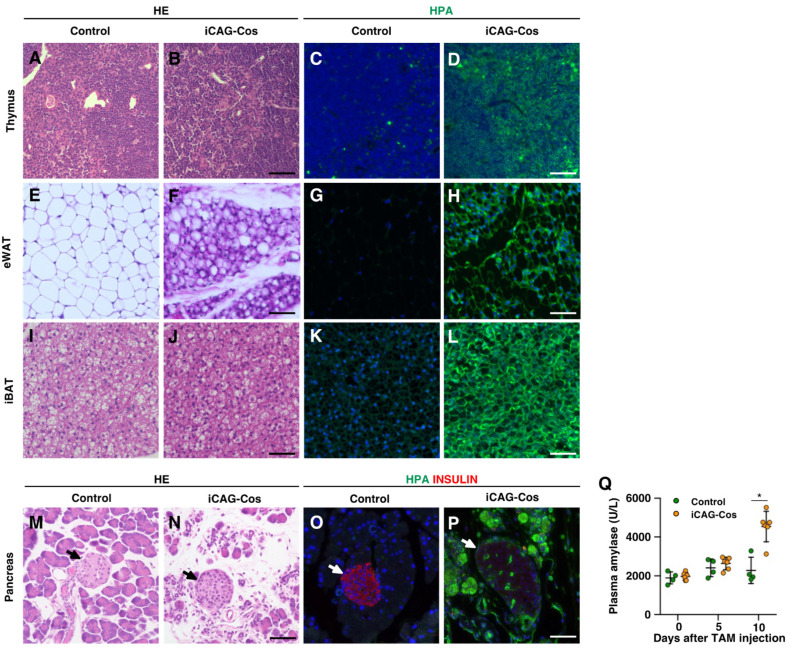
Histological analysis of the thymus, adipose tissues, and pancreas. HE and HPA staining of the thymus (**A**–**D**), eWAT (**E**–**H**), and iBAT (**I**–**L**) on day 10. HPA (green), and Hoechst (blue) staining is shown. (**M**,**N**) HE staining of the pancreas on day 10. (**O**,**P**) Fluorescent staining of insulin (red), HPA (green), and Hoechst (blue) in the pancreas on day 10. Black and white arrows indicate pancreatic islets. (**Q**) Measurement of plasma amylase activity on days 0, 5, and 10 in control and iCAG-Cos mice. Control, *n* = 4; iCAG-Cos, *n* = 6; scale bar = 50 µm, * *p* < 0.05. iBAT, interscapular brown adipose tissue; eWAT, epididymis white adipose tissue.

First, we analyzed organs that exhibited decreased weights on day 10. The relative organ weight of the thymus in iCAG-Cos mice was reduced to approximately half of that of the control (control, 0.18 ± 0.04; iCAG-Cos, 0.09 ± 0.02; Figure 1G). Hematoxylin–eosin (HE) staining of the thymus showed that it had a normal appearance, but most of the thymic cells were HPA-positive (Figure 2A–D). T-cell-specific *Cosmc*-KO mice, induced with Lck-Cre mice, have been reported to have a 20% decrease in total thymic cell numbers compared to that in wild-type animals [13]. In iCAG-Cos mice, there was a 50% decrease in the thymic weight compared to the control weight, and thus, those differences might be due to the effects of abnormalities in tissues other than the thymus. Although the abnormalities in other organs are described later, thymic regression is known to be caused by various stresses; for example, it has been reported that oxidative stress and increased inflammation due to nephrotoxicity can cause thymic involution [15]. For iCAG-Cos mice, this is thought to be caused by a decrease in T cell numbers due to defects in core 1-derived *O*-glycans in the T cells themselves and stress from multiple organ failure.

The relative organ weights of eWAT and iBAT also decreased (eWAT: control, 0.55 ± 0.05; iCAG-Cos, 0.24 ± 0.03; iBAT: control, 0.29 ± 0.01; iCAG-Cos, 0.17 ± 0.03; Figure 1G). HE staining of eWAT and iBAT showed atrophy of adipocytes in iCAG-Cos mice (Figure 2E,F,I,J). In addition, adipocytes in these tissues of iCAG-Cos mice showed a greater intensity of HPA, which indicates that both eWAT and iBAT have core 1-derived *O*-glycan modified proteins (Figure 2G,H,K,L). Then, we generated adipocyte-specific *Cosmc*-KO mice (Adipoq-Cos) by mating *Cosmc*-floxed mice with Adipoq-Cre mice. Although HPA staining showed altered glycan structures in both eWAT and iBAT of Adipoq-Cos mice, adipocyte atrophy was not observed wih HE staining, which implied that the atrophy of adipocytes seen in iCAG-Cos mice was due to the failure of other organs (Supplementary Figure S1A–H).

Since the pancreas in iCAG-Cos mice showed a significant decrease in the relative organ weight (control, 1.40 ± 0.10; iCAG-Cos, 0.61 ± 0.05), we performed HE staining on the pancreas. The extensive loss of pancreatic acinar cells and expansion of intracellular space were observed in the iCAG-Cos mice, whereas cells from the control mice had a normal appearance (Figure 2M,N). To confirm the structural changes in core 1-derived *O*-glycans in iCAG-Cos mice, pancreatic sections were stained using HPA lectin. Pancreatic acinar cells of iCAG-Cos mice were strongly stained with HPA compared to those of control mice. However, the intensity of the HPA signal in pancreatic islets was unchanged between the control and iCAG-Cos mice, and the expression of insulin as a pancreatic β cell marker was decreased in iCAG-Cos mice (Figure 2O,P). Plasma amylase activity was measured as a marker of pancreatitis, and iCAG-Cos mice showed a significant increase in blood amylase activity on day 10 (control, 2284 ± 340 U/L; iCAG-Cos, 4548 ± 318 U/L; Figure 2Q), suggesting that the loss of core 1-derived *O*-glycans caused acute pancreatitis. A previous study reported that a mouse model of pancreatitis caused by ligation of the bile duct showed decreased numbers of CD4 + and CD8 + cells in the thymus [16]. Pancreatitis observed in iCAG-Cos mice might thus contribute to thymic atrophy. Moreover, pancreatic acinar cells assist in digestion and absorption by secreting digestive enzymes such as amylase, lipase, and trypsin into the duodenum. It is possible that the atrophy of pancreatic acinar cells caused nutritional disturbances and increased lipid metabolism, resulting in the atrophy of adipocytes. In fact, the blood glucose level of iCAG-Cos mice was much lower than that of control mice (control: 11.88 ± 0.51 mmol/L, iCAG-Cos: 7.34 ± 0.55 mmol/L, Table 1). Since the 12 h fasting blood glucose levels of control mice was 6.51 ± 0.23 mmol/L (*n* = 6), iCAG-Cos mice are considered to have lower blood glucose levels, similar to those in a state of fasting. From a paper on fasting mice, it was inferred that fasting for 2~3 days causes atrophy of adipocytes to the level seen in iCAG-Cos mice [17]. Therefore, the low nutritional status of iCAG-Cos mice might contribute to the atrophy of adipocytes.

Next, to investigate whether acute pancreatitis observed in iCAG-Cos mice was directly caused by the loss of core 1-derived *O*-glycans in pancreatic acinar cells, we generated pancreas-specific *Cosmc*-KO mice (Pdx1-Cos) by mating *Cosmc*-floxed mice with mice that express Cre under control of the *Pdx1* promoter. Atrophy of pancreatic acinar cells was not observed in 8-week-old Pdx1-Cos mice, and pancreatic islets were also found to be intact (Supplementary Figure S2A,B). However, HPA staining of the pancreas in Pdx1-Cos mice showed HPA-reactive cells in both endocrine and exocrine cells (Supplementary Figure S2C–F). In addition, HPA with insulin or glucagon co-staining indicated that pancreatic α cells and β cells harbored core 1-derived *O*-glycans. These data suggest that the acute and severe pancreatitis observed in iCAG-Cos mice was caused by the deteriorative effects from other tissues and that the decreased expression of insulin in iCAG-Cos mice is not due to the loss of core 1-derived *O*-glycans.

The systemic and inducible loss of Wilms tumor 1 (*Wt1*) using CAG-CreER^TM^ has been reported in mice that exhibit multiple organ failure, including the atrophy of pancreatic exocrine cells similar to that in iCAG-Cos mice, and *Wt1*-deficient mice induced by Pdx1-Cre also do not show the atrophy of pancreatic exocrine cells [18]. In this paper, the authors suggested that the loss of *Wt1* activates pancreatic satellite cells to secrete inflammatory cytokines and chemokines, resulting in pancreatitis. Whether pancreatic satellite cells have core 1-derived *O*-glycan needs to be investigated; however, it is possible that pancreatic satellite cells, in which the loss of *Cosmc* is not induced by Pdx1-Cre, are associated with the development of pancreatitis in iCAG-Cos mice. Moreover, it has been reported that the number of zymogen granules in pancreatic exocrine cells specifically increases in pancreatic acinar cell-specific *Cosmc*-deficient mice, and these mice do not exhibit acute pancreatitis [19]. It is possible that pancreatitis is caused by a combination of the activation of digestive enzymes in the granules due to the abnormal accumulation of zymogen granules and other factors. For example, since hypertriglyceridemia is known to be a factor that increases the risk of acute pancreatitis [20], increased blood triglycerides in iCAG-Cos mice might have contributed to the induction of acute pancreatitis in iCAG-Cos mice (Table 1).

### 2.3. iCAG-Cos Mice Show the Presence of Spontaneous Gastric Ulcers

**Figure 3:**
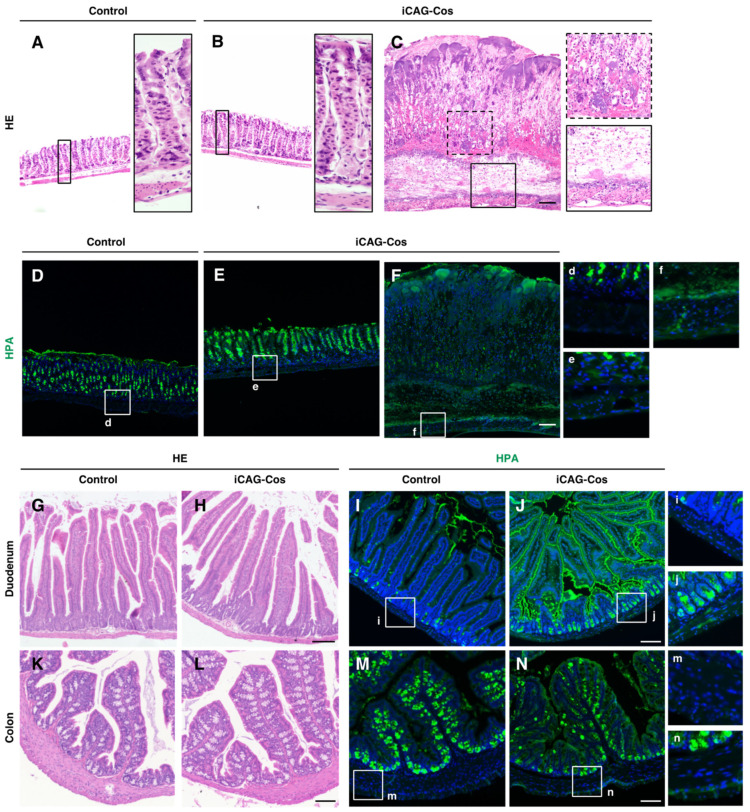
Histological analysis of digestive organs. HE and HPA staining of control stomachs (**A**,**D**), normal stomachs of iCAG-Cos mice (**B**,**E**), and stomachs of iCAG-Cos mice with gastric ulcers (**C**,**F**) on day 10. HE and HPA staining of the duodenum (**G**–**J**) and colon (**K**–**N**) on day 10. HPA (green), Hoechst (blue); scale bar = 100 µm. (**d**–**f**,**i**,**j**,**m**,**n**) Right panels show high-magnification images of the field indicated by boxes in the left panel.

The gastric tissue on day 10 in four of the nine iCAG-Cos mice showed thickening of the gastric epithelium, extensive hemorrhaging, and inflammatory infiltration into the muscle layer and serosa, which indicates the presence of severe gastric ulcers (Figure 3A–C). The gastric ulcers observed in iCAG-Cos mice were considered one of the causes of their high mortality observed in our study. This result was similar to that of a previous study showing that spontaneous gastritis is induced by the loss of core 1-derived *O*-glycans in a gastric epithelial cell-specific manner in mice [21]. However, based on the results of HPA staining, the control mice were found to have some endogenous HPA-positive cells, but the entire stomach tissue of iCAG-Cos mice was found to be HPA-positive. These data indicate that cells other than epithelial cells in the stomach also possess core 1-derived *O*-glycans (Figure 3D–F).

The duodenum and colon in iCAG-Cos mice were seen to be normal in appearance on day 10 (Figure 3G,H,K,L). HPA staining showed the loss of core 1-derived *O*-glycans in epithelial cells, as well as other cells comprising the duodenum and colon (Figure 3I,J,M,N). In the duodenum, we observed an increase in the number of HPA-positive cells in the intestinal glands, and a stronger HPA signal was observed in the luminal epithelium in iCAG-Cos mice compared to that in control mice. In the colon, as in the small intestine, a stronger HPA, compared to that in the control group, was observed in luminal epithelial cells. However, weaker HPA signals were observed in colon goblet cells of iCAG-Cos mice compared to those in controls. One study using Villin-CreER^T2^ by Fu et al. showed a decrease in the thickness of the mucosal layer 5–10 days after core 1-derived *O*-glycan deficiency induction [22]. In addition, the intensity of Alcian blue staining, which stains the mucosa, and the protein expression of Muc2 and Muc3, which are carrier proteins of core 1-derived *O*-glycans that constitute the mucus, were also decreased. Therefore, we speculated that the decreased mucosa production and the decreased amount of mucus carrier proteins in iCAG-Cos mice might have resulted in a decrease in the staining of goblet cells by HPA. The core 3 structure of GlcNAc-β1,3-GalNAc-α-Ser/Thr, is one of the core structures of mucin-type *O*-glycans, which is synthesized by a unique enzyme, core 3 β1,3-*N*-acetylglucosaminyltransferase (B3gnt6) [23]. B3gnt6 synthesizes the core 3 structure by transferring GlcNAc to GalNAc of the Tn antigen. Since both the core 1 and core 3, structures are synthesized from Tn antigens, the biosynthesis of the core 1 structure competes with the synthesis of the core 3 structure. In addition, it has been reported that *B3gnt6* is selectively expressed in the salivary glands and small and large intestines [24]. In a previous study, *B3gnT6*-KO mice were crossed with intestinal epithelial cell-specific *C1galt1*-KO mice to evaluate the role of core 1 and core 3 structures in the duodenum. *C1galt1*::*Bgnt6* double KO mice exhibited spontaneous duodenal inflammation that led to tumorigenesis [25]. This result, showing that the duodenum is unaffected by the deletion of *Cosmc* in iCAG-Cos mice, implies that the loss of core 1-derived *O*-glycans is not by itself capable of causing duodenal inflammation by itself. The deficiency of *C1galt1*, *B3gnt6*, or both in intestinal epithelial cells disrupts the colorectal environment and causes spontaneous colitis and tumorigenesis [22,24,26]. Owing to the high mortality of iCAG-Cos mice, we analyzed mice on day 10. Considering previous reports, we infer that the number of days between induction and analysis might have been insufficient for the infiltration of inflammatory cells into the colon.

Previous studies on the function of core 1 and core 3 structures in the digestive tract have focused on gastrointestinal epithelial cells. In this study, we found that cells other than epithelial cells, such as the muscle layer and serosa in the gastrointestinal tract, also possess core 1-derived *O*-glycans. Future studies on the function of core 1-derived *O*-glycans in other structures are required.

### 2.4. iCAG-Cos Mice Show Renal Injury with Albuminuria

**Figure 4:**
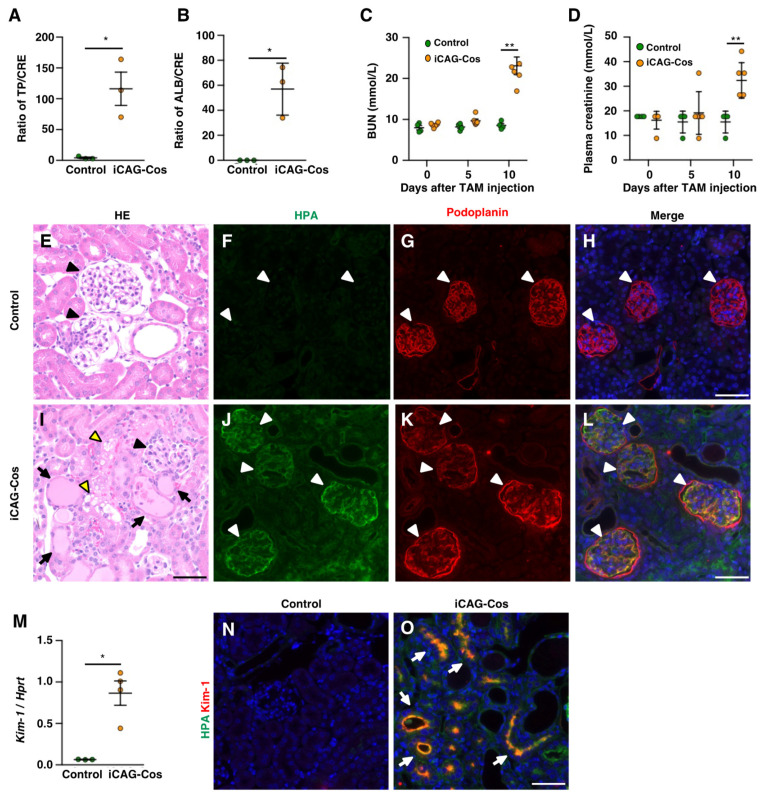
Histological and biochemical analyses of the kidney. (**A**,**B**) Quantification of urine total proteins (TP)/creatinine (CRE) ratio and urine albumin (ALB)/CRE ratio in control and iCAG-Cos mice (*n* = 3) on day 10. (**C**,**D**) Measurement of BUN and creatinine on day 0, 5, and 10 in control and iCAG-Cos mice. (Control, *n* = 4; iCAG-Cos, *n* = 5). HE staining and of fluorescent staining of HPA lectin (green), podoplanin (red), and Hoechst (blue) in kidneys of control (**E**–**H**) and iCAG-Cos (**I**–**L**) mice on day 10. Black and white arrowheads indicate glomeruli. Black arrows and yellow arrowheads indicate protein casts and necrotic renal tubules, respectively. (**M**) Relative transcript levels of *Kim-1* on day 10. *Hprt* was used for normalization. (**N**,**O**) Immunostaining image of Kim-1 (red) with HPA (green). White arrows indicate Kim-1 positive renal tubules. Scale bar = 50 µm, * *p* < 0.05, ** *p* < 0.01.

Previously, we reported that glomerular epithelial cell (podocyte)-specific *Cosmc*-KO mice (iPod-Cos) showed impairments in the glomerular filtration barrier [8]. As expected, the urine total protein (TP)/creatinine (CRE) and urine albumin (ALB)/CRE ratios were significantly elevated in iCAG-Cos mice on day 10 (TP/CRE: control, 4.29 ± 1.26; iCAG-Cos, 116.14 ± 27.07; ALB/CRE: control, 0.06 ± 0.03; iCAG-Cos, 57.04 ± 12.00; Figure 4A,B). A significant increase in blood urea nitrogen (BUN) and plasma CRE on day 10 compared to those in the controls were also seen in iCAG-Cos mice (BUN: control, 8.54 ± 0.46 mmol/L; iCAG-Cos, 23.10 ± 2.15 mmol/L; CRE: control, 15.47 ± 2.21 mmol/L; iCAG-Cos, 32.41 ± 2.95 mmol/L; Figure 4C,D). In addition, protein casts were broadly observed by HE staining of cells in iCAG-Cos mice on day 10, which showed the presence of some necrotic renal tubular cells (Figure 4E,I). These data indicate that the glomerular filtration function was impaired in iCAG-Cos mice. To confirm changes in the core 1-derived *O*-glycan structures in the kidney, kidney sections were stained using HPA lectin and anti-podoplanin antibodies as podocyte markers. A higher intensity of HPA in iCAG-Cos mice was observed in glomeruli and renal tubules, whereas the kidney in the controls showed a very low intensity of HPA in these cells (Figure 4F–H,J–L). In the glomeruli of iCAG-Cos mice, HPA-reactive and anti-podoplanin antibody-reactive cells were co-localized, indicating the presence of core 1-derived *O*-glycans in podocytes. Moreover, the urine TP/CRE ratio in iCAG-Cos mice was significantly elevated compared to that in iPod-Cos mice (iCAG-Cos, 116.14 ± 27.07; iPod-Cos, 43.11 ± 0.65; *p* < 0.05) [8], suggesting that core 1-derived *O*-glycans expressed in kidney cells other than podocytes are also important for maintaining kidney functions. Moreover, our previous study using glomerular epithelium cell-specific *Mafb* KO (Mafb-cKO) mice with segmental glomerulosclerosis showed the death of mice even with a relatively mild impairment in renal function compared to that in the iCAG-Cos mice [27]. The values of each index in Mafb-cKO and iCAG-Cos mice are as follows: U-TP/CRE: Mafb-cKO, 52.04 ± 9.65; iCAG-Cos, 116.14 ± 27.07; U-ALB/CRE: Mafb-cKO, 23.29 ± 5.65; iCAG-Cos, 57.04 ± 12.00; creatinine: Mafb-cKO, 25.64 ± 2.65 mmol/L; iCAG-Cos, 32.41 ± 2.95 mmol/L; BUN: Mafb-cKO, 13.03 ± 0.79 mmol/L; iCAG-Cos, 23.10 ± 2.15 mmol/L. These results suggested that the severe kidney dysfunction observed in iCAG-Cos mice contributed to their high mortality.

Kidney injury molecule 1 (Kim-1, also known as Tim-1, a protein from the T cell immunoglobulin and mucin domain (Tim) gene family), is a membrane-bound glycoprotein expressed in the proximal renal tubules. It has been reported that its expression is elevated with the occurrence of renal tubular injury [28,29]. Considering the possibility that renal tubular injury contributes to severe kidney dysfunction in iCAG-Cos mice, we measured the expression of *Kim-1* in kidney samples. The relative expression of the *Kim-1* transcript was significantly increased in iCAG-Cos mice compared to that in the controls (Figure 4M). Immunohistochemical analysis revealed that Kim-1 was not expressed in the controls but was found to be localized to the lumens of renal tubules in iCAG-Cos mice (Figure 4N,O). These data implied that the renal tubules in the iCAG-Cos mice were damaged. Interestingly, Kim-1 has a mucin domain with 32 putative mucin-type *O*-glycosylation sites [11], and loss of the mucin domain in Kim-1 has been reported to exacerbate renal tubular injury caused by acute kidney injury [30]. In addition, we previously reported that core 1-derived *O*-glycans on Tim-4, a member of the Tim gene family, are important for increasing the stability of Tim-4 [6]. These studies suggest that the loss of core 1-derived *O*-glycan on Kim-1 might enhance the severity of renal tubular injury. Further studies are required to elucidate the function of core 1-derived *O*-glycans in renal tubules.

Serological analysis indicated that total protein in the plasma was decreased in iCAG-Cos mice (Table 1). It was thought that this low blood protein level was caused by excessive protein release into the urine. iCAG-Cos mice also showed elevated plasma triglyceride levels (Table 1). Some reports have shown that the discharge of blood albumin into urine due to kidney dysfunction leads to increases in blood triglyceride levels and low-density lipoprotein [31]. Urinary albumin levels were increased in the iCAG-Cos mice (Figure 4B). Kidney impairment observed in iCAG-Cos mice is a possible cause of elevated blood triglyceride levels. We also performed HE and HPA staining of other tissues (Supplementary Figure S3). The appearance of these tissues in iCAG-Cos mice was normal, although all organs showed elevated reactivity against HPA. However, it is possible that the loss of core 1-derived *O*-glycans might affect the function of these tissues. For example, an in vitro study using the hepatocellular carcinoma cell line HepG2 showed that the loss of core 1-derived *O*-glycans on low-density lipoprotein receptors (LDLRs) decreases the interaction between LDLR and LDL [32]. Although this function of core 1-derived *O*-glycans has not been examined in vivo, the deletion of *Cosmc* in hepatocytes might be considered to contribute to the dyslipidemia exhibited by iCAG-Cos mice (Supplementary Figure S3).

In other studies, mice with inducible and global deficiency of core 1-derived *O*-glycans have also been generated by crossing *C1galt1* flox mice with Rosa26-rtTA; tetO-Cre Tg mice [7]. These mice showed kidney dysfunction as well, but survived for more than 8 weeks after the induction of *C1galt1* deletion. Moreover, mice harboring a point mutation in *C1galt*1 had low C1galt1 activity, thrombocytopenia, and proteinuria [3]. The mice in this study also showed decreased mortality compared to that in iCAG-Cos mice. However, these studies did not report thymic atrophy, the atrophy of adipose tissues, acute pancreatitis, gastric ulcers, and dyslipidemia, which are phenotypes observed in iCAG-Cos mice. These studies and our study differ in the efficiency and timing of the deletion of genes related to the biosynthesis of core 1-derived *O*-glycans. This difference might lead to different phenotypes even in adults.

## 3. Materials and Methods

### 3.1. Animals

Mice were maintained in specific pathogen-free conditions at the Laboratory Animal Resource Center at the University of Tsukuba, Ibaraki, Japan. The generation of mice harboring the floxed *Cosmc* allele was performed using a method described in our previous study [6]. *Cosmc*^flox/flox^ mice were crossed with B6.Cg-Tg(CAG-cre/Esr1*)5Amc/J (CAGCre-ER^TM^) transgenic mice [33] to generate ubiquitous tamoxifen (TAM)-inducible *Cosmc*-KO mice (CAGCre-ER^TM^::*Cosmc^flox/flox^*; iCAG-Cos). Conditional excision of the floxed allele of 6-week-old mice was achieved via the intraperitoneal injection of TAM (75 mg/kg body weight) once daily for 5 consecutive days. The day of the final TAM injection was defined as day 0. As control mice for iCAG-Cos mice, TAM-induced CAGCre-ER^TM^ negative, *Cosmc^flox/Y or flox^* mice were used. Tg(Pdx1-Cre)89.1Dam/Mmcd (Pdx1-Cre) mice and B6;FVB-Ta(Adipoq-Cre)1Evdr/J (Adipoq-Cre) mice were purchased from the Jackson Laboratory (Bar Harbor, Maine, USA). As control mice for Pdx1-Cre and Adipoq-Cre mice, Cre negative, *Cosmc^flox/Y or flox^* mice were used.

### 3.2. Helix Pomatia Agglutinin Analysis

The *Helix pomatia* agglutinin (HPA) blot analysis was conducted using tissues ground in liquid nitrogen and resuspended in lysis buffer (50 mM Tris-HCl (pH 7.4), 150 mM NaCl, 1 mM EDTA, 1% Triton X-100) containing cOmplete^TM^ protease inhibitor cocktail (Roche, Basel, Switzerland). Tissue extracts were separated using SDS polyacrylamide gel electrophoresis (10% gel) and transferred to an Immobilon P membrane (Merck Millipore, Burlington, MA, USA). After blocking with Carbo-Free Blocking Solution (Vector Laboratories, Burlingame, CA, USA), the membrane was incubated with primary HRP-conjugated HPA (EY Laboratories, Inc., San Mateo, CA, USA) at 4 °C overnight. The blot was developed using Immobilon Western Chemiluminescent HRP substrate (Merck Millipore) and visualized using the iBright Imaging System (Thermo Fisher Scientific, Waltham, MA, USA). CBB staining was performed using Quick-CBB Plus according to the manufacturer’s protocol (FUJIFILM Wako, Chuo, Osaka, Japan).

### 3.3. Urine Analysis

Murine urine was collected during spontaneous micturition. The measurement of urinary total protein (pyrogallol red protein assay), creatinine (enzymatic method), and albumin (immunonephelometry) levels was carried out by Oriental Yeast Co., Ltd. (Itabashi, Tokyo, Japan).

### 3.4. Serological Analysis

Mouse plasma was collected from the inferior vena cava using heparin as an anticoagulant. Blood chemical analysis was conducted using the Fuji Dri-Chem 7000V (FUJIFILM Wako) clinical chemistry analyzer to measure levels of BUN, creatinine, triglycerides, total cholesterol, high-density lipoprotein (HDL), total protein, blood glucose and total bilirubin, as well as the activity of plasma amylase, aspartate aminotransferase (AST), and alanine aminotransferase (ALT) according to the manufacturer’s instructions.

### 3.5. Histological Analysis

Tissues were fixed in Mildform 10N (FUJIFILM Wako) overnight at 4 °C and paraffinized. Sections of 3 µm thickness were assessed after HE staining under a light microscope. For immunofluorescence analysis, sections were deparaffinized and incubated with Dako target retrieval solution (Agilent Technologies, Santa Clara, CA, USA) buffer at 110 °C for 10 min in an autoclave. After blocking with Carbo-Free Blocking Solution for 1 h, sections were incubated overnight at 4 °C with primary antibodies or lectin. Alexa Fluor conjugated secondary antibodies (Invitrogen, Waltham, MA, USA) were used for immunodetection. The details regarding the antibodies and lectins used are described in Table S1. Nuclei were counterstained with Hoechst 33,342 (Thermo Fisher Scientific, Waltham, MA, USA). Images were captured using a BIOREVO BZ-X800 microscope system (Keyence, Higashiyodogawa, Osaka, Japan).

### 3.6. Quantitative Analysis of Transcripts Using RT-qPCR

Total RNA was extracted from the kidneys using ISOGEN (NIPPON GENE, Chiyoda, Tokyo, Japan). First-strand cDNA was synthesized using the QuantiTect Reverse Transcription Kit (QIAGEN, Hulsterweg, Venlo, The Netherlands). PCR was performed using a THUNDERBIRD SYBR qPCR system (Toyobo, Kita, Osaka, Japan) and a TP850 Thermal Cycler Dice Real Time System (Takara-bio, Kusatsu, Shiga, Japan). The primer sequences for *Kim-1* and *Hprt* were as follows: *Kim-1* forward, 5′-TCCACACATGTACCAACATCAA-3′; *Kim-1* reverse, 5′-GTCACAGTGCCATTCCAGTC-3′; *Hprt* forward, 5′-CAAACTTTGCTTTCCCTGGT-3′; *Hprt* reverse; 5′-CAAGGGCATATCCAACAACA-3′. Relative amounts of *Kim-1* transcripts were normalized to the amount of *Hprt* transcripts.

### 3.7. Statistical Analysis

All data are presented as the mean ± SEM. Two-tailed *t*-tests were performed to determine statistical significance for Figure 1C–G and Figure 4A,B,M, and Table 1. For the analysis of Figure 2Q and Figure 4C,D, Tukey’s test was performed. All statistical analyses were performed using Prism 7 (GraphPad Software, Inc., San Diego, CA, USA) software with the significance level set to *p* < 0.05.

## Figures and Tables

**Table 1:** Summary of serological analysis.

Inspection Item	Control (*n* = 9)	iCAG-Cos (*n* = 11)	*p*-Value
Glucose (mmol/L)	11.88 ± 0.51	7.34 ± 0.55	0.00
Triglyceride (mmol/L)	0.71 ± 0.11	2.04 ± 0.45	0.02
Total cholesterol (mmol/L)	1.06 ± 0.04	4.22 ± 0.58	0.00
HDL ^1^ (mg/dL)	0.81 ± 0.08	2.64 ± 0.31	0.01
Total protein (g/L)	35.01 ± 1.55	28.80 ± 0.16	0.02
AST ^2^ (U/L)	28.67 ± 1.49	42.55 ± 6.19	0.05
ALT ^3^ (U/L)	24.33 ± 2.60	33.77 ± 1.41	0.01
Total bilirubin (µmol/L)	5.12 ± 0.32	4.97 ± 0.69	0.85

^1^ HDL: High-density lipoprotein; ^2^ AST: aspartate aminotransferase; ^3^ ALT: alanine aminotransferase (ALT).

## Data Availability

Data is contained within the article and Supplementary Materials. Additional details available upon request.

## Supplementary Materials

The following are available online at https://www.mdpi.com/article/10.3390/ijms23031273/s1.

## References

[1] Ju T., Brewer K., D’Souza A., Cummings R.D., Canfield W.M. (2002). Cloning and expression of human core 1 beta1,3-galactosyltransferase. J. Biol. Chem..

[2] Ju T., Cummings R.D. (2002). A unique molecular chaperone Cosmc required for activity of the mammalian core 1 beta 3-galactosyltransferase. Proc. Natl. Acad. Sci. USA.

[3] Alexander W.S., Viney E.M., Zhang J.G., Metcalf D., Kauppi M., Hyland C.D., Carpinelli M.R., Stevenson W., Croker B.A., Hilton A.A. (2006). Thrombocytopenia and kidney disease in mice with a mutation in the C1galt1 gene. Proc. Natl. Acad. Sci. USA.

[4] Wang Y., Jobe S.M., Ding X., Choo H., Archer D.R., Mi R., Ju T., Cummings R.D. (2012). Platelet biogenesis and functions require correct protein O-glycosylation. Proc. Natl. Acad. Sci. USA.

[5] Kudo T., Sato T., Hagiwara K., Kozuma Y., Yamaguchi T., Ikehara Y., Hamada M., Matsumoto K., Ema M., Murata S. (2013). C1galt1-deficient mice exhibit thrombocytopenia due to abnormal terminal differentiation of megakaryocytes. Blood.

[6] Wakui H., Fuseya S., Suzuki R., Shimbo M., Okada R., Hamada M., Kuno A., Hagiwara K., Sato T., Narimatsu H. (2018). Incomplete clearance of apoptotic cells by core 1-derived O-glycan-deficient resident peritoneal macrophages. Biochem. Biophys. Res. Commun..

[7] Song K., Fu J., Song J., Herzog B.H., Bergstrom K., Kondo Y., McDaniel J.M., McGee S., Silasi-Mansat R., Lupu F. (2017). Loss of mucin-type O-glycans impairs the integrity of the glomerular filtration barrier in the mouse kidney. J. Biol. Chem..

[8] Fuseya S., Suzuki R., Okada R., Hagiwara K., Sato T., Narimatsu H., Yokoi H., Kasahara M., Usui T., Morito N. (2020). Mice lacking core 1-derived O-glycan in podocytes develop transient proteinuria, resulting in focal segmental glomerulosclerosis. Biochem. Biophys. Res. Commun..

[9] Zeng J., Eljalby M., Aryal R.P., Lehoux S., Stavenhagen K., Kudelka M.R., Wang Y., Wang J., Ju T., von Andrian U.H. (2020). Cosmc controls B cell homing. Nat. Commun..

[10] Lowenthal M.S., Davis K.S., Formolo T., Kilpatrick L.E., Phinney K.W. (2016). Identification of Novel N-Glycosylation Sites at Noncanonical Protein Consensus Motifs. J. Proteome. Res..

[11] Kuchroo V.K., Umetsu D.T., DeKruyff R.H., Freeman G.J. (2003). The TIM gene family: Emerging roles in immunity and disease. Nat. Rev. Immunol..

[12] Linden S.K., Sutton P., Karlsson N.G., Korolik V., McGuckin M.A. (2008). Mucins in the mucosal barrier to infection. Mucosal. Immunol..

[13] Cutler C.E., Jones M.B., Cutler A.A., Mener A., Arthur C.M., Stowell S.R., Cummings R.D. (2019). Cosmc is required for T cell persistence in the periphery. Glycobiology.

[14] Li Y., Fu J., Ling Y., Yago T., McDaniel J.M., Song J., Bai X., Kondo Y., Qin Y., Hoover C. (2017). Sialylation on O-glycans protects platelets from clearance by liver Kupffer cells. Proc. Natl. Acad. Sci. USA.

[15] Betjes M.G. (2020). Uremia-Associated Ageing of the Thymus and Adaptive Immune Responses. Toxins.

[16] Ueno K., Ajiki T., Watanabe H., Abo T., Takeyama Y., Onoyama H., Kuroda Y. (2004). Changes in extrathymic T cells in the liver and intestinal intraepithelium in mice with obstructive jaundice. World J. Surg..

[17] Tang H.N., Tang C.Y., Man X.F., Tan S.W., Guo Y., Tang J., Zhou C.L., Zhou H.D. (2017). Plasticity of adipose tissue in response to fasting and refeeding in male mice. Nutr. Metab..

[18] Chau Y.Y., Brownstein D., Mjoseng H., Lee W.C., Buza-Vidas N., Nerlov C., Jacobsen S.E., Perry P., Berry R., Thornburn A. (2011). Acute multiple organ failure in adult mice deleted for the developmental regulator Wt1. PLoS Genet..

[19] Wolters-Eisfeld G., Mercanoglu B., Hofmann B.T., Wolpers T., Schnabel C., Harder S., Steffen P., Bachmann K., Steglich B., Schrader J. (2018). Loss of complex O-glycosylation impairs exocrine pancreatic function and induces MODY8-like diabetes in mice. Exp. Mol. Med..

[20] Mentula P., Kylanpaa M.L., Kemppainen E., Puolakkainen P. (2008). Obesity correlates with early hyperglycemia in patients with acute pancreatitis who developed organ failure. Pancreas.

[21] Liu F., Fu J., Bergstrom K., Shan X., McDaniel J.M., McGee S., Bai X., Chen W., Xia L. (2020). Core 1-derived mucin-type O-glycosylation protects against spontaneous gastritis and gastric cancer. J. Exp. Med..

[22] Fu J., Wei B., Wen T., Johansson M.E., Liu X., Bradford E., Thomsson K.A., McGee S., Mansour L., Tong M. (2011). Loss of intestinal core 1-derived O-glycans causes spontaneous colitis in mice. J. Clin. Investig..

[23] Iwai T., Inaba N., Naundorf A., Zhang Y., Gotoh M., Iwasaki H., Kudo T., Togayachi A., Ishizuka Y., Nakanishi H. (2002). Molecular cloning and characterization of a novel UDP-GlcNAc:GalNAc-peptide beta1,3-N-acetylglucosaminyltransferase (beta 3Gn-T6), an enzyme synthesizing the core 3 structure of O-glycans. J. Biol. Chem..

[24] An G., Wei B., Xia B., McDaniel J.M., Ju T., Cummings R.D., Braun J., Xia L. (2007). Increased susceptibility to colitis and colorectal tumors in mice lacking core 3-derived O-glycans. J. Exp. Med..

[25] Gao N., Bergstrom K., Fu J., Xie B., Chen W., Xia L. (2016). Loss of intestinal O-glycans promotes spontaneous duodenal tumors. Am. J. Physiol. Gastrointest. Liver Physiol..

[26] Bergstrom K., Fu J., Johansson M.E., Liu X., Gao N., Wu Q., Song J., McDaniel J.M., McGee S., Chen W. (2017). Core 1- and 3-derived O-glycans collectively maintain the colonic mucus barrier and protect against spontaneous colitis in mice. Mucosal. Immunol..

[27] Usui T., Morito N., Shawki H.H., Sato Y., Tsukaguchi H., Hamada M., Jeon H., Yadav M.K., Kuno A., Tsunakawa Y. (2020). Transcription factor MafB in podocytes protects against the development of focal segmental glomerulosclerosis. Kidney Int..

[28] Vaidya V.S., Ozer J.S., Dieterle F., Collings F.B., Ramirez V., Troth S., Muniappa N., Thudium D., Gerhold D., Holder D.J. (2010). Kidney injury molecule-1 outperforms traditional biomarkers of kidney injury in preclinical biomarker qualification studies. Nat. Biotechnol..

[29] Moresco R.N., Bochi G.V., Stein C.S., De Carvalho J.A.M., Cembranel B.M., Bollick Y.S. (2018). Urinary kidney injury molecule-1 in renal disease. Clin. Chim. Acta.

[30] Yang L., Brooks C.R., Xiao S., Sabbisetti V., Yeung M.Y., Hsiao L.L., Ichimura T., Kuchroo V., Bonventre J.V. (2015). KIM-1-mediated phagocytosis reduces acute injury to the kidney. J. Clin. Investig..

[31] Chan D.T., Dogra G.K., Irish A.B., Ooi E.M., Barrett P.H., Chan D.C., Watts G.F. (2009). Chronic kidney disease delays VLDL-apoB-100 particle catabolism: Potential role of apolipoprotein C-III. J. Lipid Res..

[32] Wang S., Mao Y., Narimatsu Y., Ye Z., Tian W., Goth C.K., Lira-Navarrete E., Pedersen N.B., Benito-Vicente A., Martin C. (2018). Site-specific O-glycosylation of members of the low-density lipoprotein receptor superfamily enhances ligand interactions. J. Biol. Chem..

[33] Hayashi S., McMahon A.P. (2002). Efficient recombination in diverse tissues by a tamoxifen-inducible form of Cre: A tool for temporally regulated gene activation/inactivation in the mouse. Dev. Biol..

