# Supplementary material for: Global Loss of Core 1-Derived O-Glycans in Mice Leads to High Mortality Due to Acute Kidney Failure and Gastric Ulcers
Source: Int J Mol Sci. 2022 Jan 24;23(3):1273. doi: 10.3390/ijms23031273 (PMC8835874; doi:10.3390/ijms23031273)
Supplement: Supplementary file 1 [file ijms-23-01273-s001.zip › Figure_S1.pdf]

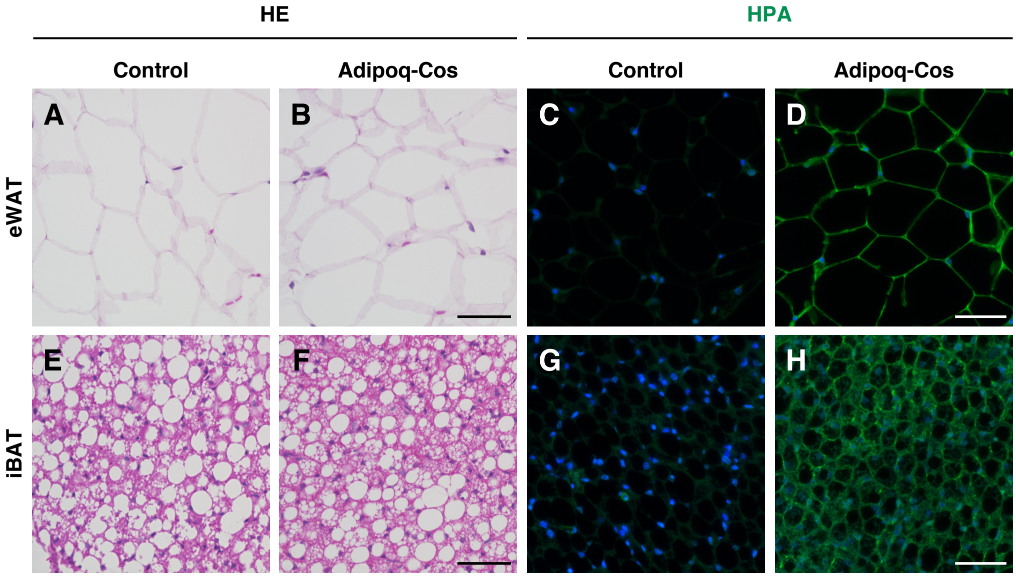


**Figure S1.** Histological analysis of adipose tissues in Adipoq-Cos mice. H&E and HPA staining of eWAT (A-D) and iBAT (E-H) in Adipoq-Cos mice. HPA (green), Hoechst (blue), Scele bar = 50 μm
