# Supplementary material for: Global Loss of Core 1-Derived O-Glycans in Mice Leads to High Mortality Due to Acute Kidney Failure and Gastric Ulcers
Source: Int J Mol Sci. 2022 Jan 24;23(3):1273. doi: 10.3390/ijms23031273 (PMC8835874; doi:10.3390/ijms23031273)
Supplement: Supplementary file 1 [file ijms-23-01273-s001.zip › Figure_S2.pdf]

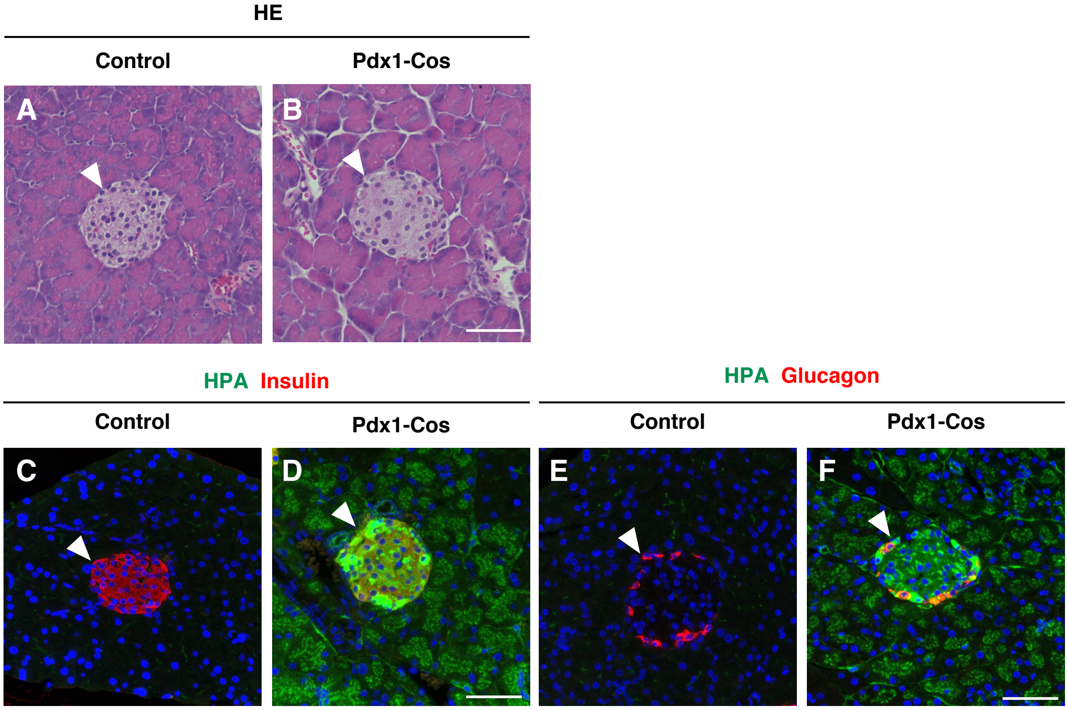

Figure S2. Histological analysis of pancreas in Pdx1-Cos mice. (A, B) H&E staining of pancreas in Pdx1-Cos mice. (C, D) Fluorescent staining of HPA (green) and insulin (red). (E, F) Fluorescent staining of HPA (green) and glucagon (red). White arrowheads indicate pancreatic islets. Scale bar = 50 μm.
