# Supplementary material for: Global Loss of Core 1-Derived O-Glycans in Mice Leads to High Mortality Due to Acute Kidney Failure and Gastric Ulcers
Source: Int J Mol Sci. 2022 Jan 24;23(3):1273. doi: 10.3390/ijms23031273 (PMC8835874; doi:10.3390/ijms23031273)
Supplement: Supplementary file 1 [file ijms-23-01273-s001.zip › Figure_S3.pdf]

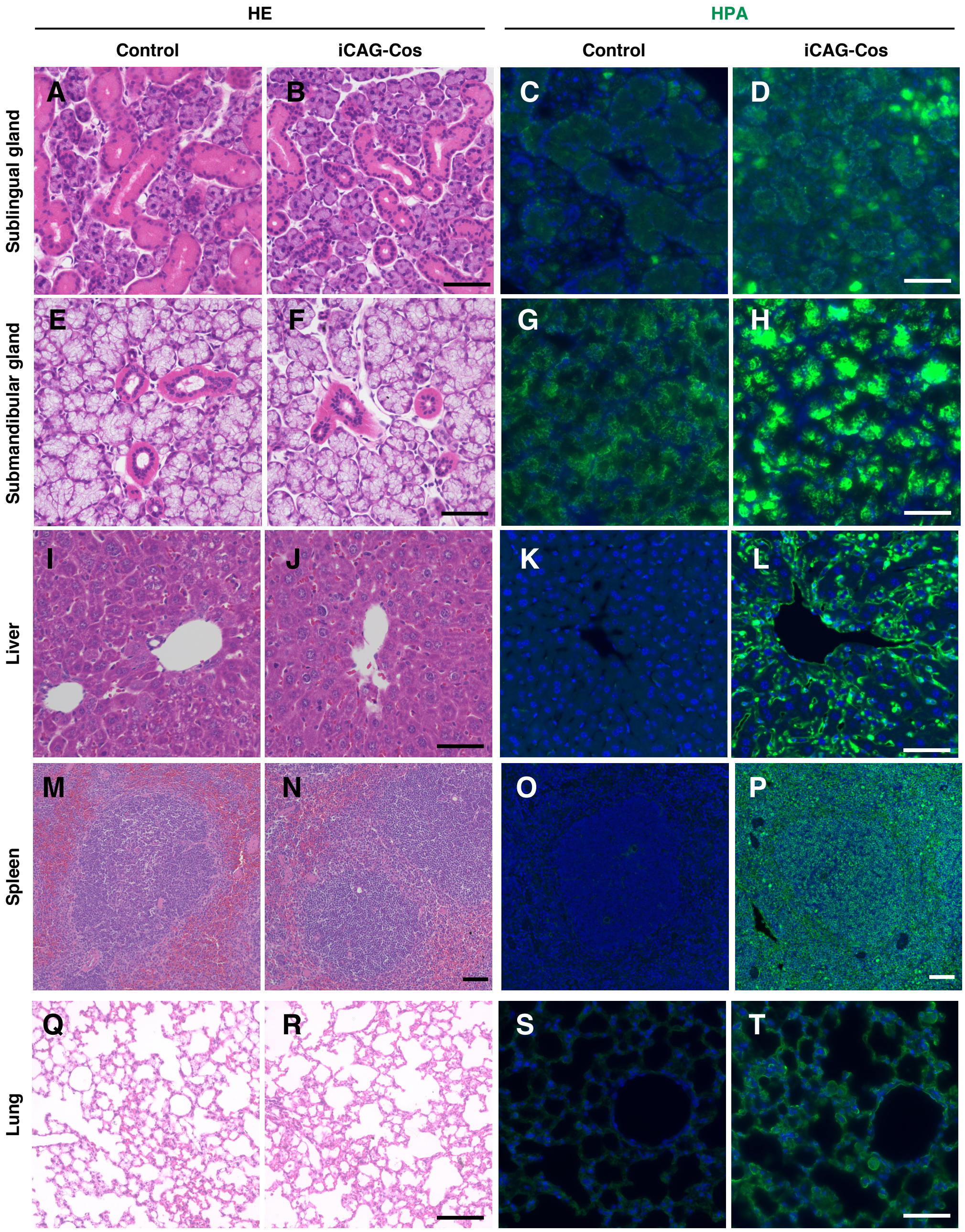


**Figure S3.** Histological analysis of other tissues. H&E and HPA staining of sublingual gland (A-D), submandibular gland (E-H), liver (I-L), spleen (M-P), and lung (Q-T) on day 10. HPA (green), Hoechst (blue), Scale bar = 50 μm.
