# Supplementary material for: Global Loss of Core 1-Derived O-Glycans in Mice Leads to High Mortality Due to Acute Kidney Failure and Gastric Ulcers
Source: Int J Mol Sci. 2022 Jan 24;23(3):1273. doi: 10.3390/ijms23031273 (PMC8835874; doi:10.3390/ijms23031273)
Supplement: Supplementary file 1 [file ijms-23-01273-s001.zip › Table_S1.pdf]

Table S1. Details of lectin and antibodies

Name	Manufacturer	Code	Dilution
Alexa Fluor 488 conjugated HPA	Thermo Fisher Scientific	L11271	1:200
Goat anti Podoplanin antibody	R&D systems	AF3244	1:20
Rat anti Kim-1 antibody	R&D systems	MKM100	1:500
Guinea pig anti-Insulin antibody	Abcam	ab7842	1:100
Rabbit anti-Glucagon antibody	Cell Signaling technology	2760	1:500
Alexa Fluor 647 conjugated Donkey anti-Rat IgG H&L antibody	Abcam	ab150155	1:1000
Alexa Fluor 594 conjugated Goat anti-Guinea pig IgG H&L antibody	Thermo Fisher Scientific	A11076	1:1000
Alexa Fluor 594 conjugated Donkey anti-Rabbit IgG H&L. antibody	Thermo Fisher Scientific	A21207	1:1000
